# Epistaxis—Its Causation and Treatment

**Published:** 1902-10

**Authors:** 


### Epistaxis—Its Causation and Treatment.

Gordon King, in *New Orleans Medical and Surgical Journal* for April, classifies the causes as: (1) Traumatism. (2) Local nasal disease. (3) Constitutional conditions. (4) Vicarious function. Under treatment are mentioned pressure, followed by cauterization of the bleeding point, the use of 870 solutions of antipyrin as a spray, etc., while great stress is laid upon the value of a solution of adrenalin 1-1000, in sodium chloride solution, this produces a local ischemic effect lasting for half an hour and upwards, during which time the point of trauma can be cauterized with electricity, silver nitrate or chromic acid. As 20 to 30 minims of this solution also acts as a powerful heart stimulant when administered internally, a combination of local and systemic treatment is often a most happy one. C. R. M.

